# Uncertain Drop vs. Socially Evaluated Cold Pressor: Uncertain Stress Elicits Stronger Psychophysiological Responses and Differential Neural Oscillatory Patterns

**DOI:** 10.3390/brainsci16050445

**Published:** 2026-04-23

**Authors:** Panhui Wang, Kewei Sun, Shengdong Ye, Di Wu, Shengli Li, Xiaodong Zhao, Wei Xiao

**Affiliations:** 1 Department of Military Medical Psychology, Air Force Medical University, Xi’an 710032, China; wangpanhui@fmmu.edu.cn (P.W.); xlxsunkewei@126.com (K.S.); 13405998279@163.com (S.Y.); xlxxwudi@fmmu.edu.cn (D.W.); 2 Shenyang Selection Center of the Air Force Recruiting Pilot Office, Shenyang 110015, China; 17691212120@163.com; 3 Unit 95926 of the PLA, Changchun 130012, China; 18792791251@163.com

**Keywords:** acute stress induction, uncertain stress, fear of falling, frequency domain analysis

## Abstract

**Highlights:**

**What are the main findings?**

- The Uncertain Drop Stress Test (UDST) effectively induced psychophysiological stress responses, with significantly stronger effects than the Socially Evaluated Cold Pressor Test (SECPT).
- UDST enhanced high-frequency (Beta/Gamma) neural activity (exogenous vigilance mode), whereas SECPT suppressed low-frequency (Theta/Alpha) activity (interoceptive focusing mode); females exhibited higher stress reactivity.

**What are the implications of the main findings?**

- UDST provides a novel, standard, and effective laboratory paradigm for inducing acute uncertain stress, with important methodological implications.
- The distinct neural patterns between certain and uncertain stress may inform interventions for stress-related disorders, particularly considering sex differences.

**Abstract:**

Objective: This study developed the Uncertain Drop Stress Test (UDST), an uncertain stress induction paradigm based on the high survival-relevant threat of fear of falling, wherein neither the occurrence nor the timing of the fall is predictable. The aim was to compare its stress induction efficacy and neural oscillatory changes with those of the Socially Evaluated Cold Pressor Test (SECPT), a certain stress paradigm, and to examine gender differences. Methods: Forty-eight participants (24 males; 24 females) were recruited. Psychological indicators (subjective stress, negative affect, and state anxiety) and physiological indicators (heart rate, heart rate variability, galvanic skin response, and salivary cortisol) were measured before and after stress to compare induction efficacy. Resting-state EEG was collected for frequency domain analysis to explore neural oscillatory changes. Results: UDST induced more pronounced psychophysiological changes. Notably, only UDST significantly decreased heart rate variability and increased galvanic skin response. UDST triggered an “exogenous vigilance mode” characterized by enhanced high-frequency (Beta/Gamma) activity, whereas SECPT elicited an “interoceptive focusing mode” characterized by suppressed low-frequency (Theta/Alpha) activity. Females exhibited higher heart rate and Beta activity than males under both stress conditions. Conclusions: UDST elicits stronger psychophysiological responses and distinct neural oscillatory patterns, with females showing greater stress reactivity.

## 1. Introduction

Stress refers to the complex neurobiological response of an individual to real or potential threatening stimuli [1]. Acute stress, typically lasting for a short duration (minutes to one month), exerts immediate effects on mental health and cognitive function, positioning it as a central focus in stress research [2]. During acute stress, individuals typically exhibit psychophysiological and neural oscillatory changes. Subjectively, emotional states are altered [3], leading to negative emotions like anxiety [4], and individuals subjectively perceive pressure and tension [5]. Objectively, activation of the sympathetic adrenal medullary (SAM) axis triggers sympathetic nervous system excitation, manifesting as increased blood pressure, heart rate, and galvanic skin response [6]. Activation of the hypothalamic–pituitary–adrenal (HPA) axis causes the adrenal cortex to release large amounts of the glucocorticoid cortisol [7]. Regarding neural oscillations, the Alpha band, associated with relaxation, often shows decreased power during stress responses, while the Beta and Gamma bands, linked to information processing and attentional functions, respectively, frequently exhibit increased power [8]. However, findings regarding power changes in the Theta band remain inconsistent [9,10].

Investigating the changes in emotion, cognition, and behavior during acute stress is of great significance, with a key challenge being the development or selection of appropriate stress induction methods. Existing paradigms for inducing acute stress are diverse, such as the Cold Pressor Test (CPT), the Trier Social Stress Test (TSST), and the Socially Evaluated Cold Pressor Test (SECPT). However, these primarily focus on certain stress, where the probability of the stressor occurring after a period is 100% (e.g., a speech is bound to happen, or cold water immersion is inevitable), with relatively few studies addressing uncertain stress [11]. Real-world environments are inherently uncertain, particularly in military contexts. For instance, novel combat forces extensively utilized in conflicts like the Russia–Ukraine war—such as drones, robotic dogs, and micro-incapacitating weapons—impose continuous psychological deterrence and cognitive load on personnel due to the high uncertainty surrounding their timing of appearance, mode of action, and lethal efficacy, greatly exacerbating fear and anxiety [12]. Therefore, although classical paradigms have been validated for standardization and effectiveness, they struggle to reflect the pervasive uncertainty in real-world environments, which limits the ecological validity of research findings in the stress field, thereby highlighting the theoretical and practical importance of conducting research on uncertain stress.

Uncertainty can be categorized into occurrence uncertainty, timing uncertainty, and intensity uncertainty [13]. Compared to certain threats, uncertain threats possess the following characteristics: (1) They also negatively impact individuals; for example, uncertainty about future threats can diminish an individual’s coping ability and trigger anxiety [14]. A study simulating air traffic controllers performing a vigilance task also found that uncertainty could trigger significant stress and impair task performance [15]. (2) They elicit different individual responses. Certain threats primarily induce fear, avoidance, and defensive aggression, whereas uncertain threats mainly induce anxiety, vigilance, and behavioral inhibition [16]. (3) Their effects on attention differ. When a threat is certain, an individual’s attention selectively focuses on the threat itself. When a threat is uncertain, attention is dispersed across the entire environment, increasing the probability of detecting the threat but at the cost of higher cognitive load [17]. (4) They are more threatening. Uncertain threats, especially those involving timing uncertainty, can induce stress and are more likely to trigger anxiety compared to certain threats [18]. Moreover, as uncertainty increases, so do the perceived pain, fear levels, and physiological arousal in individuals [19].

Existing studies have used an unpredictable electric shock threat as an uncertain stressor. Electric shock poses both a physiological threat to an individual’s comfort and health and a psychological threat through anticipatory anxiety generated by its unpredictability [7]. Some studies have found that unpredictable electric shock significantly increases subjective anxiety and decreases heart rate variability [20]. However, the efficacy of electric shock in inducing stress is debated: some research has shown no significant increase in salivary cortisol concentrations under shock conditions, indicating ineffective HPA axis activation [21]; other studies combining virtual reality with electric shock threat found only significant heart rate increases, with no significant changes in salivary alpha-amylase or cortisol [22]. Furthermore, electrode stimulation intensity is limited by ethical and safety thresholds, hindering further improvements in stress induction strength and ecological validity. Therefore, there is an urgent need to develop a new acute stress induction paradigm that not only captures the characteristics of uncertainty but also effectively activates both the SAM and HPA axes.

From an evolutionary perspective, survival threat relevance is a key factor influencing stress intensity. Survival threat relevance refers to the likelihood that a specific stimulus has consistently posed a threat throughout human evolutionary history. Compared to threat types emerging in modern society, stimuli with long-standing evolutionary bases for threat tend to evoke more intense fear responses [23]. Fear of falling has evolved into an inherent survival instinct and protective mechanism throughout human evolution. The sudden sensation of weightlessness induces intense unease, closely linked to perceptions of losing control and facing danger. Compared to relatively modern threats like electric shock, falling possesses higher survival threat relevance, constituting a genuine existential threat and serving as a high-intensity psychophysiological stressor. For example, the Heidelberg Risk Sport-Specific Stress Test (HRSST), which uses falling as a stressor by requiring participants to jump from a 12 m high rock wall, experiencing a 3 m drop, has been shown to effectively induce state anxiety [24].

Introducing an uncertainty variable into the falling process aligns with the concept of uncertain stress and may achieve more ideal stress induction effects. Accordingly, this study developed a novel method for inducing acute uncertain stress—the Uncertain Drop Stress Test (UDST). UDST leverages the high survival-relevant stimulus of falling, combined with timing and occurrence uncertainty, creating an unpredictable falling scenario where both the occurrence and timing of a drop are unknown, thereby simulating real-world uncertain stress environments. UDST involves both the physiological stimulation of the fall threat and the psychological stimulation arising from uncertainty.

Although UDST has theoretical advantages, its actual stress induction efficacy has not yet been systematically validated, and to date, no study has directly compared the psychophysiological and neural oscillatory changes induced by uncertain versus certain stress. Furthermore, many studies on gender differences in stress recruit only male participants to control for hormonal influences, resulting in a lack of sufficient empirical evidence regarding female stress response characteristics and gender differences in the field. In fact, males and females exhibit differential responses to stress. Males typically display a “fight-or-flight” response [25], whereas females are additionally influenced by the menstrual cycle. For instance, elevated estrogen can activate the parasympathetic nervous system, leading to a “tend-and-befriend” response [26]. Beyond stress responses, males and females also show activation differences at the neural oscillation level. Wriessnegger et al. [27], using a mental arithmetic task combined with noise and negative feedback to induce stress, found that females showed significantly increased Theta and Beta activity in the prefrontal cortex, while males exhibited more pronounced increases in Gamma power.

In summary, this study aims to compare the stress induction efficacy and neural oscillatory changes elicited by the Uncertain Drop Stress Test (UDST) and the Socially Evaluated Cold Pressor Test (SECPT), a classic acute stress induction paradigm also combining psychological and physiological stimulation. By measuring psychological indicators (subjective stress, negative affect, and state anxiety) and physiological indicators (heart rate, heart rate variability, galvanic skin response, and salivary cortisol) before and after stress, changes in the SAM and HPA axes were assessed. Resting-state EEG was collected for frequency domain analysis to explore neural oscillatory changes. Potential gender differences were also analyzed, aiming to provide empirical evidence for understanding the differential stress effects and neural mechanisms underlying uncertain versus certain stress.

## 2. Materials and Methods

### 2.1. Participants

A three-factor mixed design was employed to compare stress induction efficacy, with gender as a between-subjects variable, and condition and time as within-subjects variables. Sample size was calculated using G*Power 3.1.9.7. Selecting repeated measures ANOVA, and based on previous research indicating that stress-induced psychophysiological changes should at least yield a medium effect size [28], the effect size was set at 0.25, with a significance level at 0.05 and power at 0.8, and two levels for the between-subjects factor and four levels for the within-subjects factors. The calculation yielded a required sample size of 24. Forty-eight healthy students were recruited from a university, including twenty-four males (mean age 21.71 ± 1.97 years) and twenty-four females (mean age 21.46 ± 2.13 years). Inclusion criteria were as follows: (1) no history of psychiatric or neurological disorders; (2) no history of heart disease or hypertension; (3) no long-term use of psychiatric, neurological, or endocrine medications; (4) no history of severe lower-limb trauma, no acute injury recently (past 6 months), and currently normal functional activity; (5) female participants were not in their menstrual period [5]. Exclusion criteria were as follows: (1) participation in stress-related experiments within the past 6 months; (2) suffering from periodontitis, oral ulcers, or gum bleeding; (3) experienced major psychological stressors within the past 2 months; (4) chronic sleep deprivation or reversed day/night schedule. Termination criterion were as follows: Participants could not tolerate the stress or withdrew midway for other reasons. To ensure accuracy of cortisol measurement, participants refrained from alcohol for 72 h and smoking for 24 h prior to the experiment, had good sleep the night before, avoided vigorous exercise and any beverages other than water on the experiment day, and fasted from food and water for 1 h before the start. All participants provided informed consent before the experiment and received compensation upon completion.

### 2.2. Stress Induction Methods

#### 2.2.1. Uncertain Drop Stress Test

##### Design Rationale

The UDST developed in this study is based on the following principles: (1) Stressor selection: Based on the theory of survival threat relevance, the fear of falling has a long evolutionary basis and has evolved into an inherent survival instinct, making it more likely to induce strong fear responses than relatively modern threats (e.g., electric shock). Therefore, the UDST uses falling as the stressor, leveraging its high survival threat relevance to achieve stronger stress induction effects. (2) Introduction of uncertainty: Based on the characteristics of uncertain threats, occurrence uncertainty and temporal uncertainty can induce stress and are more likely to trigger anxiety than certain threats. Accordingly, this study introduced an uncertain falling context in which whether and when a drop will occur are both unknown. The introduction of uncertainty is a core component of the UDST stressor, and by simulating the uncertain real-world environment it helps to improve the ecological validity of the stress induction method. (3) Operationalization of uncertainty: A standardized set of instructions creates an uncertain falling context for participants. Although the drop time points are pre-fixed to ensure experimental consistency, participants are completely unaware of this schedule. Thus, although the drop sequence is objectively fixed, what participants experience is genuine subjective uncertainty (including both occurrence and temporal uncertainty)—precisely the psychological state of uncertain anticipation that the UDST aims to manipulate. (4) Determination of key parameters: Regarding the trapdoor opening, the dimensions of 100 cm × 80 cm ensure that participants do not hit the edges when falling. Regarding drop height, the 75 cm setting is primarily constrained by laboratory ceiling height and safety considerations, providing sufficient stress intensity while ensuring safety under laboratory conditions. Regarding the number of trials, a total of three trials are set, each lasting a maximum of 2 min. Three trials provide sufficient opportunities to operationalize uncertainty, while keeping the total stress duration within 6 min, which is informed by the duration design of classic stress induction paradigms—ensuring adequate stress accumulation while avoiding reduced stress responses due to excessive fatigue or habituation. Regarding drop timings, the three drop time points are pre-fixed as 30 s after standing onset, 90 s after standing onset, and no drop. This timing parameter is based on pilot data, which showed that during the 2 min standing period, participants‘ anxiety levels under uncertain anticipation exhibited two peaks at approximately 30 s and 90 s. The absence of a drop in the third trial serves to break participants’ expectation that “a drop will occur on every trial,” thereby maintaining uncertain anticipation throughout the stress procedure, while also meeting the requirement of a 4 min resting-state EEG acquisition duration.

##### Drop Apparatus

**Figure 1:**
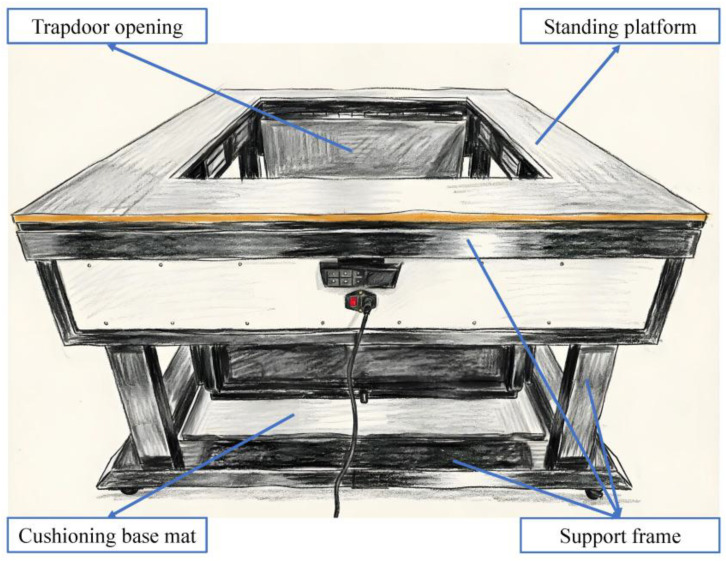
Schematic diagram of the drop device.

A trapdoor drop method suitable for laboratory settings was adopted, where participants stood on a platform, and stress was induced by controlling the opening of a trapdoor to cause a sudden drop. The drop apparatus was self-developed, as shown in Figure 1, and included the following core components: (1) Support frame, the main structure of the apparatus, consisting of a bottom, a top, and four support columns, with dimensions of 140 cm × 120 cm × 75 cm (length × width × height). (2) Standing platform, fixed above the top support frame, made of a 2 cm-thick polyethylene board, with the surface covered by foam padding to ensure participant safety. (3) Trapdoor opening, located in the middle of the standing platform, measuring 100 cm × 80 cm, connected and fixed to the top support frame via stainless steel hinges; the trapdoor was controlled by four magnetic locks, which instantly opened upon receiving an “open” signal from a remote control, causing a sudden drop, and remained closed upon receiving a “close” signal to allow safe standing. (4) Cushioning base mat, located at the bottom of the support frame, providing cushioning protection.

##### Induction Procedure

**Figure 2:**
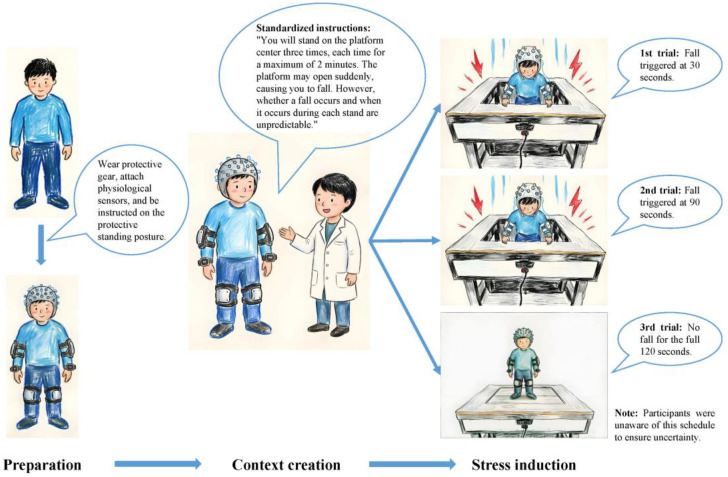
UDST stress process.

The specific induction procedure was as follows: (1) Preparation: Participants were fitted with safety gear, the required physiological sensors were attached, and participants were instructed on the protective standing posture: feet slightly apart, knees slightly bent, arms slightly flexed. (2) Context creation: Standardized instructions were read to create an uncertain threat context: “You will stand on the platform center three times, each time for a maximum of 2 min. The platform may open suddenly, causing you to fall. However, whether a fall occurs and when it occurs during each stand are unpredictable.” This effectively manipulated occurrence uncertainty and timing uncertainty. To ensure experimental consistency, the first fall was triggered at 30 s, the second at 90 s, and no fall occurred during the third 120 s stand. Participants were unaware of this schedule to maintain uncertainty. The purpose of including a no-fall trial was to prevent participants from predicting whether a fall would occur on the third trial based on the pattern of the first two falls, thereby maintaining an uncertain expectation throughout the experiment. (3) Stress induction: With the trapdoor “closed,” the participant was guided to stand on the platform center, and the timer for the first trial was started. At the pre-set, unpredictable time, the trapdoor was “instantaneously opened” causing a brief, safe drop of 75 cm. The trapdoor was then “closed,” and the participant was guided back to the platform center for the second trial. This procedure was repeated until the third trial concluded. The specific stress process is shown in Figure 2.

#### 2.2.2. Socially Evaluated Cold Pressor Test

Before the test, participants were informed that they would undergo a “tolerance test” and were required to sit upright in a chair, completely immersing their right hand in ice water (0–4 °C) and trying to hold it there for 4 min. During the test, a video camera located 30 cm directly in front of them would record their facial expressions throughout—participants were told that the video would be used for subsequent facial expression coding analysis—while one male and one female researcher sitting in front of them, maintaining neutral and serious expressions, would hold a standardized rating sheet, record the participant’s behavior and tolerance performance every 30 s, and make brief notes on the rating sheet. If participants found the procedure truly unbearable, they could stop at any time.

### 2.3. Experimental Materials

In this study, the following four categories of measures were selected to comprehensively assess different dimensions of the stress response: (1) subjective measures (subjective stress, negative affect, state anxiety) were used to assess the psychological experience dimension of stress; (2) heart rate, heart rate variability, and electrodermal activity were used to assess the rapid response of the sympathetic–adrenal–medullary (SAM) axis; (3) salivary cortisol was used to assess the delayed response of the hypothalamic–pituitary–adrenal (HPA) axis; and (4) electroencephalography (EEG) band power was used to assess oscillatory changes in the central nervous system. These four categories of measures correspond to different levels of the stress response and together constitute a multidimensional assessment system ranging from subjective to objective and from peripheral to central.

#### 2.3.1. Psychophysiological Indicator Collection

##### Subjective Stress Report

A 5-point scale was used to assess the participant’s pressure and tension level at a given moment: 1 = very relaxed; 2 = relatively relaxed; 3 = between tense and relaxed; 4 = relatively tense; 5 = very tense [29].

##### Negative Affect Scale

The Negative Affect Schedule (NAS) from the Positive and Negative Affect Schedule (PANAS) was used, consisting of 10 items rated on a 5-point scale, where higher scores indicate more negative emotions [30].

##### State Anxiety Inventory

The State Anxiety Inventory (SAI) from the State Trait Anxiety Inventory (STAI) was used, comprising 20 items rated on a 4-point scale, with higher scores indicating higher levels of state anxiety [31].

##### Heart Rate and Heart Rate Variability Acquisition

A heart rate strap (Polar H10, Polar Electro Oy, Kempele, Finland) was used to record heart rate (HR) and heart rate variability (HRV) at a sampling rate of 130 Hz, paired with a Polar heart rate monitor watch (Polar Pacer Pro, Polar Electro Oy, Kempele, Finland) worn on the right wrist. The strap was fastened around the chest [32]. HRV refers to the variation in time intervals between consecutive heartbeats, reflecting the activity and balance of the sympathetic and parasympathetic nervous systems [33]. The indicators used and their meanings are shown in Table 1.

##### Galvanic Skin Response Acquisition

A Huixin wrist-worn wearable neurophysiological measurement device (Psychorus, Huixin, Beijing, China) was used to collect Galvanic Skin Response (GSR) via four metal sensors on the back of the watch case, sampled at 40 Hz, worn on the left wrist. GSR includes Skin Conductance Level (SCL), the baseline conductance without specific environmental stimuli, and Skin Conductance Response (SCR), related to stimulus events [34]. The indicator used was the integration of SCRs (iSCR), representing the area under the curve of the skin conductance response (time integral).

##### Salivary Cortisol Acquisition

Dedicated saliva collection tubes were used. Participants were asked to passively drool (letting saliva pool on the tongue’s floor and then transfer into the tube) to collect 1 mL of saliva, which was stored in a −20 °C freezer [35].

#### 2.3.2. Resting-State EEG Measurement

**Figure 3:**
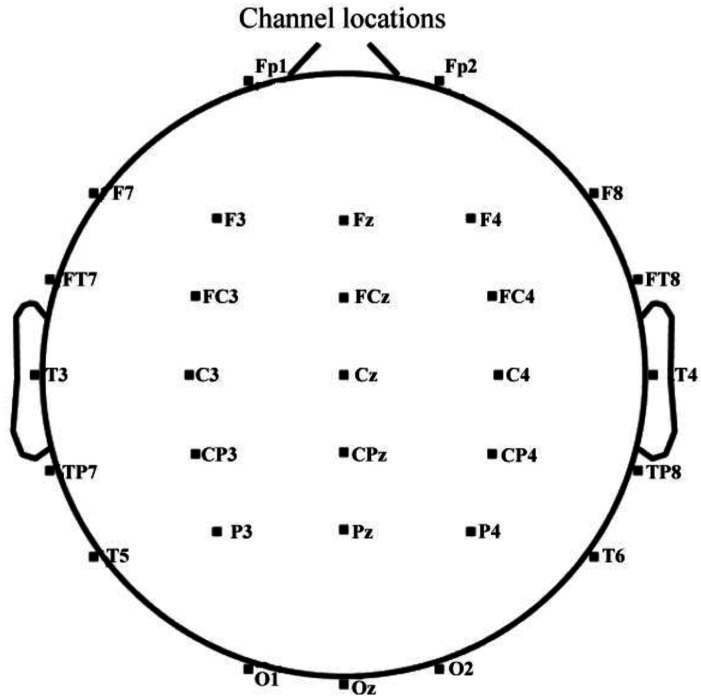
Distribution of recording electrode positions.

A wireless multi-channel EEG acquisition system (ZhenTec NT1, Xi’an ZhenTec Technology Co., Ltd., Xi’an, China) was used. A 32-channel EEG cap was placed according to the international 10-10 system, including 30 recording electrodes (positions shown in Figure 3) and 2 electrooculography (EOG) electrodes. These electrodes featured absorbent sponges pre-moistened with 3% sodium chloride solution. Electrode sites included Fp1, Fp2, F3, F4, F7, F8, Fz, FCz, FC3, FC4, FT7, FT8, C3, C4, CPz, CP3, CP4, Cz, T3, T4, T5, T6, TP7, TP8, P3, P4, Pz, O1, O2, and Oz. EEG data were sampled at 500 Hz, with CPz as the reference electrode and FPz as the ground. Impedance between electrodes and scalp was maintained below 20 kΩ. Each participant underwent recording of over 4 min of eyes-open resting-state EEG signals under three conditions: resting baseline, post-SECPT, and post-UDST. During recordings, participants were instructed to minimize head, facial, and eye movements, keep their eyes open focusing on a wall marker (natural blinking allowed), concentrate, and remain awake throughout. For the UDST condition, markers were placed manually before each fall and after regaining stable standing to facilitate subsequent data segment removal.

### 2.4. Experimental Procedure

**Figure 4:**
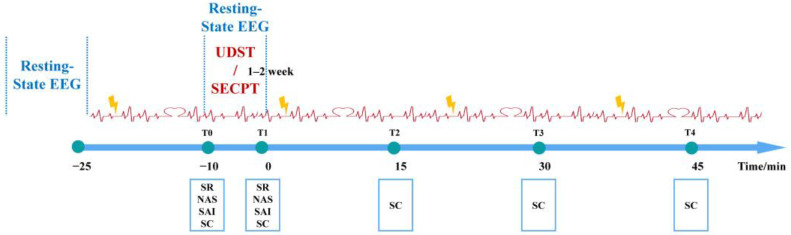
Experimental Procedure Flowchart. Note: SR = Subjective Stress Report; NAS = Negative Affect Scale; SAI = State Anxiety Inventory; SC = Salivary Cortisol; 0 min = immediately post-stress.

To mitigate carryover effects on cortisol secretion, the two stress methods were administered one week apart (no more than two weeks) [36]. To counterbalance the order of the two stress methods, male and female participants were randomly assigned numbers 01–24 using Excel. Odd-numbered participants (*N* = 12 per gender) underwent UDST first, while even-numbered participants (*N* = 12 per gender) underwent SECPT first. To control for the circadian rhythm of salivary cortisol, experimental sessions for each participant started at one of three consistent times daily: 13:00, 15:00, or 17:00. Before their first stress session, each participant completed the resting-state EEG measurement under resting conditions. Subsequently, they came to the lab on two separate occasions to complete the UDST and SECPT procedures. Upon arrival for a stress session, participants were first fitted with the Polar watch, strap, and Huixin wrist device. They then rested in a waiting room for 15 min while ECG and GSR were continuously recorded. After the rest period (T0), they completed the Subjective Stress Report, NAS, and SAI questionnaires, and provided a saliva sample. They were then escorted to the laboratory for the UDST or SECPT procedure, during which resting-state EEG was collected under the stress condition. Immediately after stress cessation (T1), participants again completed the Subjective Stress Report, NAS, and SAI, and provided a saliva sample. Additional saliva samples were collected 15 min (T2), 30 min (T3), and 45 min (T4) post-stress. Throughout the experiment, participants completed the Subjective Stress Report twice, NAS twice, SAI twice, and provided saliva samples five times. HR, HRV, and GSR were recorded continuously, with timestamps marked on the heart rate monitor at T0 and T1. The experimental procedure is shown in Figure 4.

### 2.5. Data Analysis

#### 2.5.1. Psychophysiological Indicator Processing

Data from HR, HRV, and GSR were screened using the following exclusion criteria: (1) more than 30% of the recording time showed no data change or values of zero; (2) data volume was less than 30% of the average. These conditions indicated insufficient contact between the device and skin, compromising data validity [37]. For GSR, valid data were obtained from 43 participants (19 males; 24 females); data from all 48 participants were valid for other indicators. Based on timestamps, HR, HRV, and GSR data were extracted and averaged for the time periods corresponding to the eight time points (T0-T4 and specific intervals for T0/T1). HR and HRV data extraction and calculation were performed using Kubios HRV Scientific software (version 4.1.1), with a medium (0.25 s) artifact correction setting. GSR data decomposition and calculation were performed using MATLAB R2022b and the Ledalab V3.2.5 toolbox [38]. Signals were first denoised using a Gaussian smoothing algorithm and down-sampled to 10 Hz. Continuous Decomposition Analysis (CDA) was then applied to decompose the signal into SCL and SCR components, with a response window of 1–4 s and a minimum amplitude threshold of 0.05 µS. iSCR was derived and further calculated as iSCR per minute to facilitate comparison across different time segments [39]. Due to the positively skewed distribution of SCR data, a logarithmic transformation (y = ln(x + 1)) was applied. Salivary cortisol concentrations were determined using Enzyme-Linked Immunosorbent Assay (ELISA).

#### 2.5.2. Resting-State EEG Processing

Preprocessing was performed using MATLAB R2022b and the EEGLAB 2022.1 toolbox [40]: after channel localization, the two EOG channels were removed, leaving 30 EEG channels for analysis. First, an FIR bandpass filter (0.1–40 Hz; order 165; Hamming window) and an IIR notch filter (48–52 Hz; order 4; quality factor 35) were applied. Re-referencing was performed using the global average reference, and the initial reference point CPz was included to maintain full data rank [41]. Bad channels were automatically identified using the following criteria: standard deviation exceeding five times the average standard deviation across all channels, or power spectral density exceeding three times the average of neighboring channels. For the UDST condition, based on the fall events recorded during the experiment (before each fall and after returning to stable standing), data segments from 500 ms before each fall onset to 500 ms after stable standing were removed to eliminate movement artifacts caused by physical perturbation. The continuous data were then segmented into 4 s non-overlapping epochs (2000 points, sampling rate 500 Hz). Epochs with peak-to-peak amplitude > 150 μV or with 30–40 Hz power exceeding twice the full-band average power were rejected after visual inspection. Independent component analysis (ICA) was performed using the extended Infomax algorithm (runica). Because re-referencing reduced the data rank to number of channels—1, and we had 30 EEG channels (effective rank = 29), principal component analysis (PCA) was applied before ICA by setting the pca parameter to 29 to address rank deficiency. Artifact components (ocular, muscle, and electrode noise) were identified using the MARA algorithm combined with manual inspection (topography, power spectrum, and time course). Participants with more than three bad channels or with less than 1 min of remaining clean data after artifact rejection were excluded, leaving 44 valid participants (22 males, 22 females). Among the four excluded participants, one had loose electrodes under the UDST condition, two had excessive motion artifacts under the UDST condition, and one had excessive EMG artifacts under the SECPT condition that could not be effectively removed by ICA. For spectral analysis, power spectral density (PSD) was calculated for the theta (4–8 Hz), alpha (8–13 Hz), beta (13–30 Hz), and gamma (30–40 Hz) bands. For each 4 s epoch, a Hamming window (2000 points) was applied, followed by a 2000-point FFT. The power spectrum (μV^2^/Hz) was computed for each epoch and then averaged across epochs to obtain the final PSD estimate, resulting in a frequency resolution of 0.25 Hz. The calculation was implemented using MATLAB’s pwelch function with the following parameters: window = hamming(2000); overlap = 0; nfft = 2000; fs = 500 Hz. PSD values from 1 to 40 Hz were extracted, and for each frequency band the PSD was averaged to obtain absolute power. The absolute power values were log10-transformed to approximate a normal distribution before statistical analysis.

#### 2.5.3. Statistical Analysis

Statistical analyses were performed using SPSS 26.0 software. Three-factor repeated measures ANOVA was used to compare induction efficacy, with Gender (Male, Female) as the between-subjects factor, and Condition (SECPT, UDST) and Time (T0, T1; for salivary cortisol analysis, T0-T5 were included) as within-subjects factors. Stress levels (subjective stress, negative affect, state anxiety, HR, HRV, GSR, salivary cortisol) served as dependent variables. Three-factor repeated measures ANOVA was also used for frequency domain data, with Gender (Male, Female) as the between-subjects factor, and Condition (Resting, SECPT, UDST) and Brain Region (Prefrontal, Sensorimotor, Temporal) as within-subjects factors. PSD for each frequency band (Theta, Alpha, Beta, Gamma) served as the dependent variable. Greenhouse–Geisser correction was applied when sphericity assumptions were violated. Post hoc multiple comparisons and simple effects analyses were corrected using the Bonferroni method. In SPSS 26.0, checking the Bonferroni option automatically outputs the corrected *p* values. All *p* values reported in the text are Bonferroni-corrected *p* values, and the significance threshold after correction was set at 0.05.

## 3. Results

### 3.1. Comparison of Induction Efficacy

#### 3.1.1. Subjective Stress

**Figure 5:**
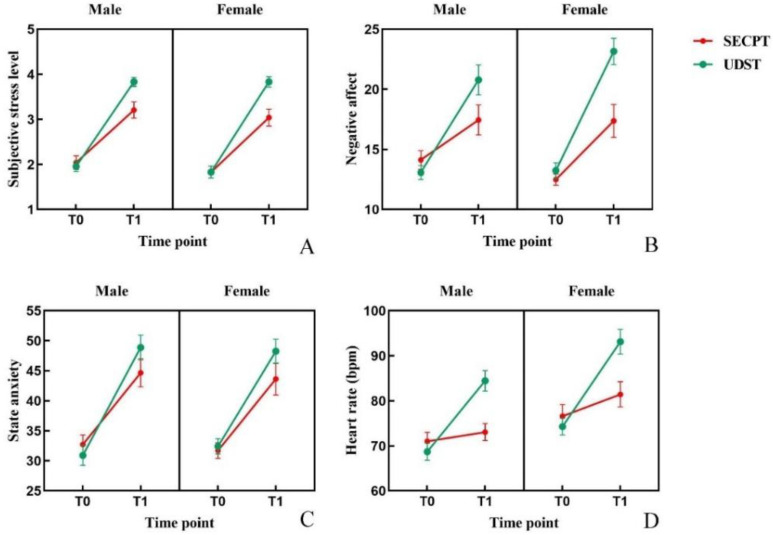
Changes in subjective scale scores and heart rate. Note: (**A**) subjective stress; (**B**) negative affect; (**C**) state anxiety; (**D**) heart rate. Error bars represent the standard error of the mean (SEM).

Scores for subjective stress across the two conditions and two time points are shown in Figure 5A. Three-factor repeated measures ANOVA revealed a significant main effect of Condition: *F*(1, 46) = 11.98; *p* = 0.001; η^2^ = 0.207. The main effect of Time was significant: *F*(1, 46) = 248.08; *p* < 0.001; η^2^ = 0.844. The Condition × Time interaction was significant: *F*(1, 46) = 18.022; *p* < 0.001; η^2^ = 0.281. Simple effects analysis showed that under both stress conditions, subjective stress at T1 was significantly higher than at T0 (*ps* < 0.001). At T1, subjective stress levels were significantly higher following UDST compared to SECPT (*p* < 0.001). The main effect of Gender and other interactions were not significant (*ps* > 0.05).

#### 3.1.2. Negative Affect

Scores for negative affect across conditions and time points are shown in Figure 5B. The main effect of Condition was significant: *F*(1, 46) = 9.647; *p* = 0.003; η^2^ = 0.173. The main effect of Time was significant: *F*(1, 46) = 118.645; *p* < 0.001; η^2^ = 0.721. The Condition × Time interaction was significant: *F*(1, 46) = 20.023; *p* < 0.001; η^2^ = 0.303. Simple effects analysis indicated that under both conditions, negative affect at T1 was significantly higher than at T0 (*ps* < 0.001). At T1, negative affect induced by UDST was significantly higher than that induced by SECPT (*p* < 0.001). The main effect of Gender and other interactions were not significant (*ps* > 0.05).

#### 3.1.3. State Anxiety

Scores for state anxiety across conditions and time points are shown in Figure 5C. The main effect of Time was significant: *F*(1, 46) = 141.352; *p* < 0.001; η^2^ = 0.754. The Condition × Time interaction was significant: *F*(1, 46) = 10.075; *p* = 0.003; η^2^ = 0.180. Simple effects analysis revealed that under both conditions, anxiety at T1 was significantly higher than at T0 (*ps* < 0.001). At T1, anxiety induced by UDST was significantly higher than that induced by SECPT (*p* = 0.012). Other main effects and interactions were not significant (*ps* > 0.05).

#### 3.1.4. Heart Rate

Changes in heart rate across conditions and time points are shown in Figure 5D. The main effect of Gender was significant: *F*(1, 46) = 5.819; *p* = 0.020; η^2^ = 0.112. The main effect of Condition was significant: *F*(1, 46) = 17.275; *p* < 0.001; η^2^ = 0.273. The main effect of Time was significant: *F*(1, 46) = 264.62; *p* < 0.001; η^2^ = 0.852. The Condition × Time interaction was significant: *F*(1, 46) = 153.980; *p* < 0.001; η^2^ = 0.770. Simple effects analysis showed that under both conditions, HR at T1 was significantly higher than at T0 (*ps* < 0.001). At T1, HR induced by UDST was significantly higher than that induced by SECPT (*p* < 0.001). The Gender × Time interaction was significant: *F*(1, 46) = 5.396; *p* = 0.025; η^2^ = 0.105. Simple effects analysis revealed that HR at T1 was significantly higher than at T0 for both genders (*ps* < 0.001); female HR at T1 was significantly higher than male HR (*p* = 0.011). Other interactions were not significant (*ps* > 0.05).

#### 3.1.5. Heart Rate Variability

**Figure 6:**
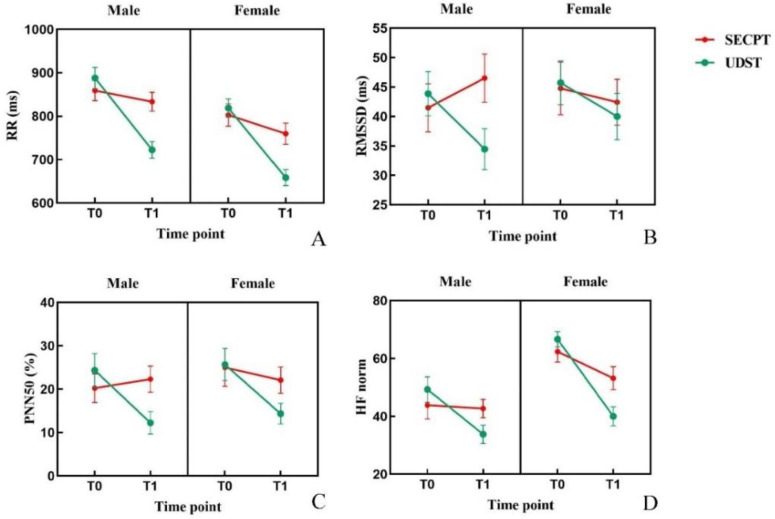
Changes in heart rate variability indicators. Note: (**A**) RR; (**B**) RMSSD; (**C**) PNN50; (**D**) HF norm. Error bars represent the standard error of the mean (SEM).

Three-factor repeated measures ANOVA showed that except for SDNN, all other HRV indicators (RR; RMSSD; PNN50; HF norm) exhibited a significant Condition × Time interaction. Simple effects analysis indicated that under UDST, these four indicators were significantly lower at T1 compared to T0 (*ps* < 0.01). However, under SECPT, only RR was significantly lower at T1 compared to T0 (*p* < 0.001). At T1, the values of these four indicators induced by UDST were significantly lower than those induced by SECPT (*ps* < 0.05). A significant main effect of Gender was found for RR (lower in females) and HF norm (higher in females). Changes in HRV indicators across conditions and time points are shown in Figure 6. Descriptive statistics and ANOVA results are presented in Table 2.

#### 3.1.6. Galvanic Skin Response

**Figure 7:**
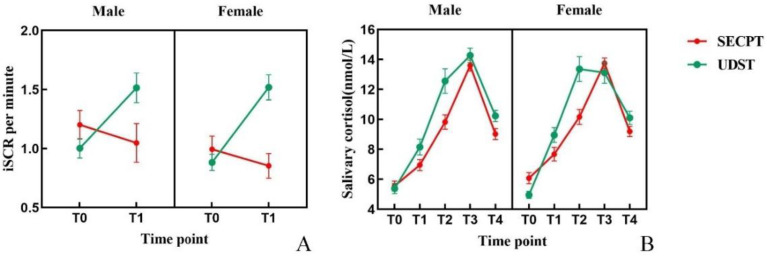
Changes in galvanic skin response and salivary cortisol. Note: (**A**) galvanic skin response (iscr); (**B**) salivary cortisol. Error bars represent the standard error of the mean (SEM).

Three-factor repeated measures ANOVA on valid GSR data (N = 43: 19 males, 24 females) revealed a significant main effect of Condition: *F*(1, 41) = 6.426; *p* = 0.015; η^2^ = 0.135. The main effect of Time was significant: *F*(1, 41) = 9.293; *p* = 0.004; η^2^ = 0.185. The Condition × Time interaction was significant: *F*(1, 41) = 17.454; *p* < 0.001; η^2^ = 0.299. Simple effects analysis showed that iSCR was significantly higher at T1 compared to T0 only under the UDST condition (*p* < 0.001). At T1, iSCR induced by UDST was significantly higher than that induced by SECPT (*p* < 0.001). The main effect of Gender and other interactions were not significant (*ps* > 0.05). Changes in iSCR across conditions and time points are shown in Figure 7A.

#### 3.1.7. Salivary Cortisol

Salivary cortisol concentrations across the five time points are shown in Figure 7B. Three-factor repeated measures ANOVA showed a significant main effect of Condition: *F*(1, 46) = 17.278; *p* < 0.00; η^2^ = 0.273. The main effect of Time was significant: *F*(4, 184) = 153.282; *p* < 0.001; η^2^ = 0.769. The Condition × Time interaction was significant: *F*(4, 184) = 10.020; *p* < 0.001; η^2^ = 0.179. Simple effects analysis revealed that under both conditions, concentration was highest at T3, significantly higher than at all other time points except T2 (*ps* < 0.001). At T1, T2, and T4, concentrations induced by UDST were significantly higher than those induced by SECPT (*ps* < 0.01). The main effect of Gender and other interactions were not significant (*ps* > 0.05).

**Figure 8:**
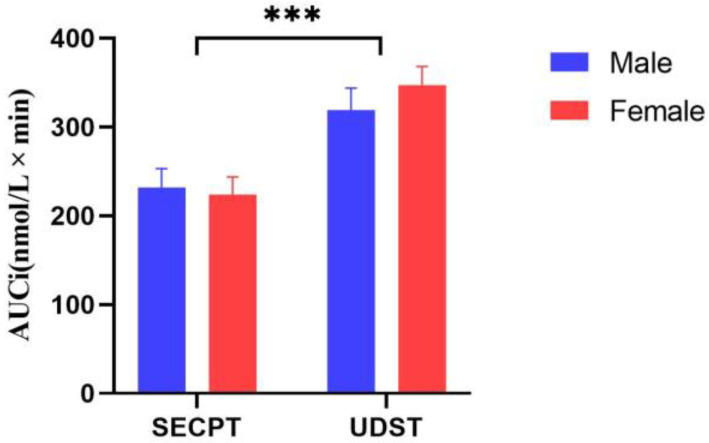
Changes in cortisol area under the curve with respect to increase (AUCi). Note: *** *p* < 0.001. Error bars represent the standard error of the mean (SEM).

To further analyze the differential cortisol reactivity induced by UDST and SECPT, a two-factor repeated measures ANOVA was performed on the area under the curve with respect to increase (AUCi). Results showed a significant main effect of Stress Method: *F*(1, 46) = 18.648; *p* < 0.001; η^2^ = 0.288, with UDST inducing significantly higher AUCi values than SECPT. The main effect of Gender and the interaction were not significant (*ps* > 0.05). Changes in cortisol AUCi are shown in Figure 8.

### 3.2. Frequency Domain Analysis Results

PSD data were extracted for Theta (4–8 Hz), Alpha (8–13 Hz), Beta (13–30 Hz), and Gamma (30–40 Hz) frequency bands. Based on brain regions closely associated with stress and areas showing color differences in topographic maps for each band, the prefrontal region (Fp1, Fp2, Fz, F3, and F4), sensorimotor region (FC3, FC4, Cz, C3, and C4), and temporal region (F7, F8, T3, T4, FT7, FT8, TP7, and TP8) were included in the analysis. The prefrontal region is a core hub for executive functions, primarily responsible for cognitive control, working memory, decision-making, and emotional regulation. The sensorimotor region is primarily involved in somatosensory integration, motor planning, and execution. The temporal region, adjacent to the amygdala and hippocampus, is involved in auditory processing, visual information, and emotional processing. After extracting PSD for each band and region, three-factor repeated measures ANOVA was conducted separately for each frequency band.

#### 3.2.1. Theta Band

**Figure 9:**
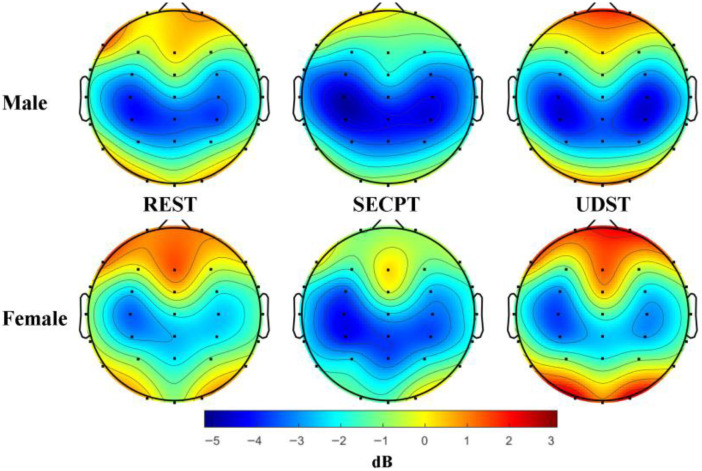
Topographic maps of theta band power spectral density. Note: Topographic maps under three conditions: rest, SECPT, and UDST stress. The unit is dB, and the color scale from blue to red indicates power from low to high. The results show that only SECPT stress induced a significant reduction in Theta power across three brain regions.

ANOVA revealed a significant main effect of Condition: *F*(2, 84) = 8.918; *p* < 0.001; η^2^ = 0.175. Post hoc comparisons showed that PSD under the SECPT condition was significantly lower than under both Resting and UDST conditions (*ps* < 0.05). This indicates that only SECPT stress caused a significant decrease in Theta band PSD. Topographic maps of Theta band PSD are shown in Figure 9.

#### 3.2.2. Alpha Band

**Figure 10:**
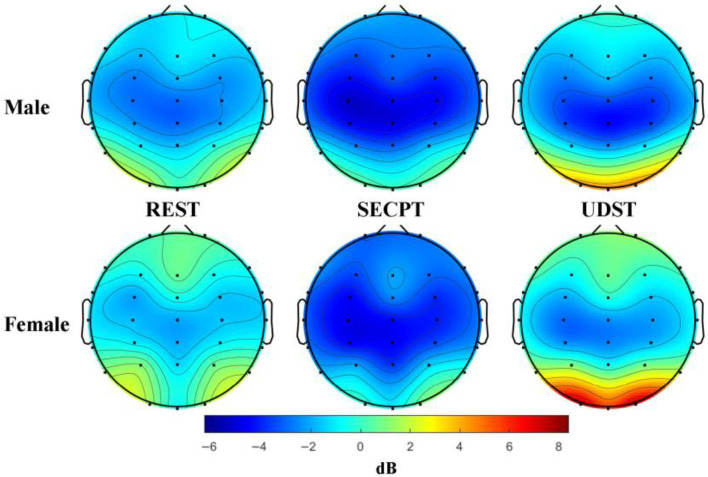
Topographic maps of alpha band power spectral density. Note: Topographic maps under three conditions: rest, SECPT, and UDST stress. The unit is dB, and the color scale from blue to red indicates power from low to high. The results show that SECPT stress induced a significant reduction in Alpha power across three brain regions in females, and a significant reduction in Alpha power in the sensorimotor region in males.

ANOVA revealed a significant main effect of Condition: *F*(2, 84) = 19.102; *p* < 0.001; η^2^ = 0.313. The Condition × Brain Region interaction was significant: *F*(4, 168) = 28.119; *p* < 0.001; η^2^ = 0.401. Simple effects analysis found that PSD induced by SECPT was significantly lower than under Resting and UDST conditions in all three brain regions (*ps* < 0.05). The Condition × Brain Region × Gender interaction was significant: *F*(4, 168) = 2.856; *p* = 0.037; η^2^ = 0.064. Simple effects analysis revealed that for females, SECPT-induced PSD was significantly lower than under Resting and UDST in all three brain regions (*ps* < 0.05). For males, this significant decrease under SECPT was observed only in the sensorimotor region (*ps* < 0.05). This indicates that only SECPT stress caused a significant decrease in Alpha band PSD, with a more widespread effect across brain regions in females. Topographic maps of Alpha band PSD are shown in Figure 10.

#### 3.2.3. Beta Band

**Figure 11:**
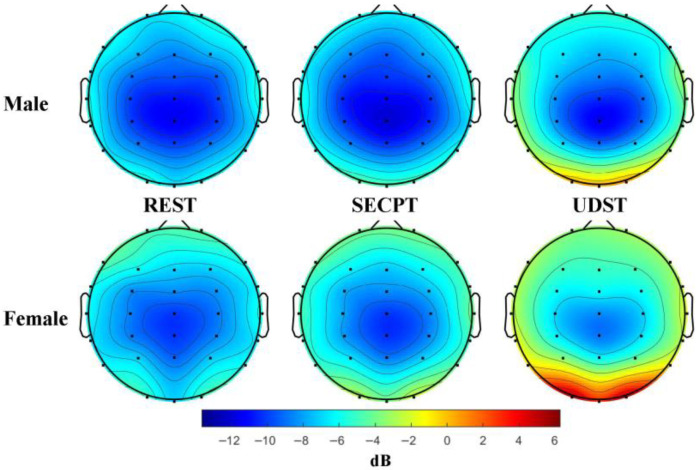
Topographic maps of beta band power spectral density. Note: Topographic maps under three conditions: rest, SECPT, and UDST stress. The unit is dB, and the color scale from blue to red indicates power from low to high. The results show that SECPT stress induced a significant increase in Beta power only in the temporal region in females, while UDST stress induced a significant increase in Beta power in the temporal region in both sexes and in the sensorimotor region in females.

ANOVA revealed a significant main effect of Condition: *F*(2, 84) = 46.162; *p* < 0.001; η^2^ = 0.524. The Condition × Brain Region interaction was significant: *F*(4, 168) = 57.264; *p* < 0.001; η^2^ = 0.577. Simple effects analysis showed that PSD induced by UDST was significantly higher than under Resting and SECPT in the sensorimotor and temporal regions (*ps* < 0.001). PSD induced by SECPT was significantly higher than under Resting only in the temporal region (*p* = 0.001). The Condition × Brain Region × Gender interaction was significant: *F*(4, 168) = 2.681; *p* = 0.033; η^2^ = 0.060. Simple effects analysis revealed that for females, UDST-induced PSD was significantly higher than under Resting and SECPT in the sensorimotor and temporal regions (*ps* < 0.01). For males, UDST-induced PSD was significantly higher than under Resting and SECPT only in the temporal region (*ps* < 0.001). SECPT-induced PSD was significantly higher than under Resting only in the female temporal region (*p* < 0.001). Females showed significantly higher PSD than males across all three brain regions under both stress conditions (*ps* < 0.05). These results indicate that UDST caused significant increases in Beta band PSD in the temporal region for both genders, with females also showing increases in the sensorimotor region. SECPT caused a significant increase only in the female temporal region. Females exhibited stronger responses to both stress types. Topographic maps of Beta band PSD are shown in Figure 11.

#### 3.2.4. Gamma Band

**Figure 12:**
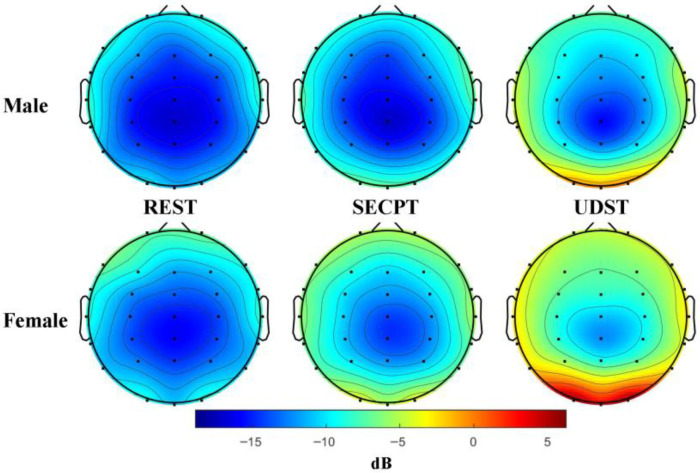
Topographic maps of gamma band power spectral density. Note: Topographic maps under three conditions: rest, SECPT, and UDST stress. The unit is dB, and the color scale from blue to red indicates power from low to high. The results show that SECPT stress induced a significant increase in Gamma power in the sensorimotor and temporal regions, while UDST stress induced a significant increase in Gamma power across three brain regions with a stronger effect.

ANOVA revealed a significant main effect of Condition: *F*(2, 84) = 86.68; *p* < 0.001; η^2^ = 0.674. The Condition × Brain Region interaction was significant: *F*(4, 168) = 76.669; *p* < 0.001; η^2^ = 0.646. Simple effects analysis showed that UDST-induced PSD was significantly higher than under Resting in all three brain regions (*ps* < 0.001), and significantly higher than SECPT in the sensorimotor and temporal regions (*ps* < 0.001). SECPT-induced PSD was significantly higher than under Resting in the sensorimotor and temporal regions (*ps* < 0.01). This indicates that both stress methods caused significant increases in Gamma band PSD in sensorimotor and temporal regions, with UDST having a stronger effect and also causing a significant increase in the prefrontal region. Topographic maps of Gamma band PSD are shown in Figure 12.

#### 3.2.5. Theta/Beta Ratio (TBR)

**Figure 13:**
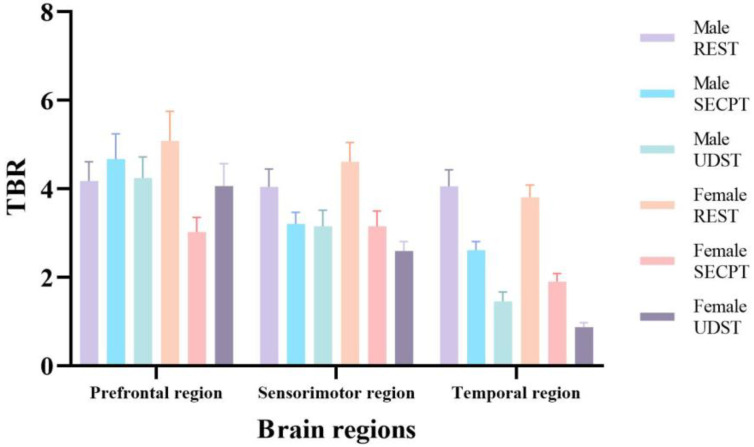
TBR results across conditions and brain regions.

TBR, the ratio of Theta to Beta power, reflects the balance between cortical inhibition and excitation, associated with cognitive control, attention regulation, and emotional arousal [42]. Increased TBR suggests a state leaning towards low arousal, internal orientation, and inhibition, while decreased TBR indicates a state leaning towards high arousal, external orientation, and excitation. Three-factor repeated measures ANOVA on TBR revealed a significant main effect of Condition: *F*(2, 84) = 24.488; *p* < 0.001; η^2^ = 0.368. The Condition × Gender interaction was significant: *F*(2, 84) = 3.544; *p* = 0.037; η^2^ = 0.078. The Condition × Brain Region interaction was significant: *F*(4, 168) = 9.894; *p* < 0.001; η^2^ = 0.191. The Condition × Brain Region × Gender interaction was significant: *F*(4, 168) = 3.164; *p* = 0.031; η^2^ = 0.070. Simple effects analysis focusing on the three-way interaction revealed that for both genders, TBR values under both stress conditions were significantly lower than under Resting in the sensorimotor and temporal regions (*ps* < 0.05). TBR values under UDST were significantly lower than under SECPT in the temporal region (*ps* < 0.001). For females, TBR values under SECPT were significantly lower than under Resting in the prefrontal region (*p* = 0.011). Females had significantly lower TBR values than males in the temporal region under both stress conditions (*ps* < 0.05). Females also had significantly lower TBR values than males in the prefrontal region under the SECPT condition (*p* = 0.016). These results indicate that both stress methods (especially UDST) caused significant decreases in TBR in sensorimotor and temporal regions for both genders, with a more pronounced decrease in the female temporal region. SECPT also caused a decrease in the female prefrontal region. TBR results across conditions and brain regions are shown in Figure 13.

## 4. Discussion

Utilizing a mixed experimental design incorporating psychophysiological assessments and resting-state EEG frequency domain analysis, this study systematically investigated the differential effects of two acute stress induction methods—the Uncertain Drop Stress Test (UDST) and the Socially Evaluated Cold Pressor Test (SECPT)—on stress responses and neural oscillations, as well as the potential moderating effects of gender.

### 4.1. Discussion of Induction Efficacy Comparison

Regarding subjective psychological indicators, both stress methods significantly elevated participants’ levels of subjective stress, negative affect, and state anxiety, with UDST inducing significantly greater changes than SECPT. This confirms that both methods effectively induce psychological stress responses, with UDST demonstrating superior efficacy. Concerning objective physiological indicators, both methods significantly increased heart rate and salivary cortisol concentrations, with UDST inducing significantly greater changes. However, regarding heart rate variability, SECPT only caused a decrease in the RR interval, whereas UDST led to significant decreases in RR, RMSSD, PNN50, and HF norm. Regarding galvanic skin response, only UDST caused a significant increase in iSCR; SECPT did not induce a significant change. These findings indicate that both methods effectively activated the HPA axis, with UDST showing stronger activation. Concerning the SAM axis, previous research has found that while cortisol increases significantly after SECPT, salivary alpha-amylase does not, suggesting suboptimal SAM axis activation [43]. Similarly, the present study found SECPT’s effects on the SAM axis to be relatively weak and limited (only HR and RR showed significant changes). In contrast, UDST effectively activated the SAM axis, demonstrating that UDST successfully induces physiological stress responses. Overall, the induction efficacy of UDST was significantly superior to that of SECPT, aligning with findings by Bradford et al. that uncertain threats, especially those involving timing uncertainty, can induce stress and are more likely to trigger anxiety than certain threats [18].

To more deeply explain the enhanced efficacy of the UDST, it is necessary to analyze it from two perspectives: the dynamic characteristics of the stressor and its evolutionary basis. From the perspective of dynamic characteristics, the SECPT provides sustained, certain physical stimulation (ice water immersion) combined with psychological pressure (social evaluation). Individuals can clearly anticipate the source and duration of the discomfort, thus primarily triggering a slow stress response mediated by the HPA axis, with relatively limited mobilization of the SAM axis. In contrast, the core feature of the UDST lies in the dual superposition of “uncertain anticipation” and “sudden drop.” For most of the time, participants are in a state of sustained anticipation of “whether and when a drop will occur.” This combination of occurrence uncertainty and temporal uncertainty continuously activates brain regions involved in anticipatory anxiety, such as the anterior cingulate cortex and insula [44], thereby maintaining a high level of sympathetic tone. The moment of the drop then provides a strong, unpredictable sudden stress event that further triggers a rapid response from the SAM axis. This biphasic structure of “sustained anticipation + sudden event” may be the neuroendocrine basis for the UDST’s more comprehensive activation of both the SAM and HPA axes. From an evolutionary perspective, falling is a genuine survival risk that human ancestors faced for a long time, possessing high survival threat relevance. Therefore, it may have a more direct and automatic neural pathway (e.g., rapidly triggering stress responses through the amygdala–hypothalamus–brainstem axis). This evolutionary “preparedness” partly explains why the UDST shows stronger effects across multiple psychophysiological indicators.

### 4.2. Discussion of Frequency Domain Analysis

Previous research indicates that stress typically leads to decreased Alpha activity and increased Beta and Gamma activity. For instance, Yang et al. [45], using a driving simulator to create stress scenarios, found significantly enhanced Beta activity and higher alertness during cognitively distracting driving compared to normal driving. Ehrhardt et al. [46] recruited 38 participants (19 females) and used the Paced Auditory Serial Addition Test (PASAT) to induce stress, finding decreased prefrontal Alpha power and increased Beta power, effects not further influenced by added time pressure or social–evaluative threat. The current study found that UDST stress elicited significant enhancements in high-frequency Beta and Gamma activities. Increased Beta power is associated with sustained cognitive vigilance, while increased Gamma power indicates heightened attention and intense emotional experience. This “high-frequency enhancement” pattern suggests that when facing uncertain psychophysiological threats, widespread brain mobilization occurs, requiring heightened environmental monitoring, preparation of somatic responses, and recruitment of cognitive resources for risk assessment and emotional processing. In contrast to UDST, SECPT stress primarily induced significant decreases in low-frequency Theta and Alpha activities. Decreased Alpha power represents cortical disinhibition, opening cortical channels for processing somatic sensations from the ice water. Decreased Theta power may reflect a reduction in task-irrelevant internal mentation. This “low-frequency suppression” pattern constitutes a state of “focused attention,” where individuals concentrate cognitive resources on enduring a sustained, certain somatic discomfort (ice water stimulation). SECPT also induced Gamma activation in sensorimotor and temporal regions, possibly related to negative emotional experiences associated with the physical discomfort. Compared to Ehrhardt et al., the lack of significant Beta increase under SECPT in this study may be because SECPT demands less cognitive effort than the PASAT.

The diametrically opposite neural oscillatory patterns revealed by the above findings highlight a fundamental difference between the two types of stress, carrying significant theoretical implications. We refer to the pattern induced by the UDST as the “exogenous vigilance mode.” The core feature of this mode is the “superimposed” mobilization of high-frequency activity—characterized by a significant increase in Beta and Gamma power without a corresponding significant decrease in fundamental low-frequency rhythms such as Theta and Alpha. This suggests that under uncertain threat, individuals do not need to suppress internally oriented processing; instead, they additionally recruit external vigilance resources while maintaining baseline brain activity. This pattern may reflect an adaptive strategy: in an unpredictable environment, individuals need to simultaneously maintain awareness of their internal state and continuously scan the external environment. In contrast, the “interoceptive focusing mode” induced by the SECPT is predominantly characterized by low-frequency suppression. This pattern is highly similar to the “focused attention” state observed in mindfulness or pain-coping research, in which individuals actively suppress activity in the default mode network and direct attentional resources toward salient somatic sensations. This aligns with the defensive behavior hierarchy model proposed by Blanchard [16]: when a threat is certain and imminent (e.g., sustained cold pain in the SECPT), individuals tend to adopt a “freezing” or “endurance” strategy—a fast, automated defensive response to cope with an ongoing somatic injury stimulus. In contrast, when the threat is uncertain (e.g., unpredictable occurrence and timing of a drop in the UDST), individuals tend to adopt a “risk assessment” strategy—continuously monitoring the environment to detect potential danger. Furthermore, both stress conditions led to significantly reduced TBR in sensorimotor and temporal regions, indicating activation of brain areas related to somatic preparation and emotional processing during stress. The significantly lower TBR in the temporal region under UDST compared to SECPT is particularly noteworthy, as the temporal region is closely linked to threat detection, uncertainty processing, and anticipatory anxiety, suggesting uncertain threats exert a stronger driving force on brain regions involved in vigilance and emotion.

Another possible alternative explanation warrants discussion: whether the drop movement in the UDST introduced motion-related artifacts that artificially elevated high-frequency power. In fact, in the present study, we rigorously removed time segments containing obvious electromyographic artifacts during the preprocessing stage, and under the UDST condition, we explicitly excluded data segments at the moment of the drop and immediately thereafter using manual markers. Therefore, the analyses primarily reflect EEG activity during the anticipatory state rather than the drop movement itself. However, we cannot completely rule out the contribution of subtle muscle tension or anticipatory electromyographic activity to the high-frequency bands. Future studies could employ independent component analysis to more finely isolate EMG artifacts. Another question worth discussing is why the UDST did not induce a significant reduction in low-frequency rhythms, as observed for the SECPT. We propose that the core stress characteristics of the UDST include both the genuine fear from the drop experience and the potential threat arising from uncertainty, with the vast majority of time spent in a state of anticipation of the uncertain threat. Although the moment of the drop itself may trigger transient low-frequency suppression, its duration is far shorter than the entire stress process; thus, it is easily masked in the averaged analysis over the whole period, resulting in the maintenance of a “preparatory mode” mediated by Beta/Gamma high-frequency activity. In contrast, SECPT induces a continuous and certain somatic sensory stimulus, requiring the cortex to remain in a “processing mode” for several minutes, thus manifesting as stable and significant low-frequency suppression.

### 4.3. Discussion of Sex Differences

At the psychophysiological level, current research on psychophysiological stress reactivity yields inconsistent conclusions. Helbig and Backhaus [47], using a public speaking task to induce stress, found that females only showed higher subjective stress reactivity than males, with no significant differences in cortisol responses. Liu and Zhang [48] found stronger physiological stress responses in males during the TSST, while Back et al. [49] reported the opposite—more pronounced changes in heart rate and cortisol for females in a TSST setting. The present study observed significantly higher stress reactivity in females only for heart rate; no significant gender differences emerged in other physiological or psychological indicators. Further research is needed to explore and validate potential gender differences in other psychophysiological measures. Furthermore, Sato and Miyake found that females exhibit higher parasympathetic activity than males [50]; Min et al. similarly confirmed that females are parasympathetically dominant [51]. In line with these findings, the present study also observed that, in terms of the main effect of sex, females had significantly higher HF norm values than males, indicating higher parasympathetic activity in females. Recent research has shown that female empathic ability is positively correlated with parasympathetic dominance but negatively correlated with sympathetic activity [52] (Menashri Sinai et al., 2024). This suggests that females’ greater ease in detecting others’ emotions and their higher tendency to exhibit empathy in social interactions may stem from autonomic nervous system differences. High sympathetic activity is associated with the “fight-or-flight” response, whereas high parasympathetic activity is associated with the “tend-and-befriend” response. Females’ lower sympathetic (fight-or-flight) tendency combined with their higher parasympathetic (tend-and-befriend) baseline together constitute a physiological foundation that is more open and sensitive to external emotional signals, though this also carries the risk of emotional over-involvement.

At the neural oscillatory level, consistent with Wriessnegger et al. [27], who found increased prefrontal Beta activity in females during a stressful mental arithmetic task with noise and negative feedback, the present study found that females exhibited significantly higher Beta activity across all three brain regions under both stress conditions (especially UDST) compared to males. This suggests a high-arousal profile in females when facing stress, meaning neural networks related to cognitive control, motor preparation, and emotional processing show higher excitability and response intensity compared to those of males. Additionally, the significantly lower TBR values in the female temporal region under both stress conditions further indicate that females are more sensitive and vulnerable to the emotional impact of stress, consistent with findings by Bianchin and Angrilli [53]. Notably, only females showed significantly increased temporal Beta activity under SECPT compared to resting. Given that SECPT was characterized overall by low-frequency suppression, this implies that while enduring the physical discomfort, females experience stronger concurrent cognitive and emotional activation. Similarly, the significantly lower prefrontal TBR in females specifically under SECPT suggests stronger activation in cognitive control and attentional resources for females. From a clinical perspective, females exhibit stronger stress reactivity at the EEG level—higher beta activity (high arousal) and lower TBR (stronger emotional engagement and cognitive control)—which may constitute an underlying neural basis for their vulnerability to developing emotion-related disorders [54].

It is worth noting that the results of sex differences in psychophysiological and neural oscillatory measures reveal cross-domain inconsistency, which may reflect three possibilities: (1) Differences in measurement sensitivity and signal-to-noise ratio between EEG and peripheral physiological indicators. EEG directly records postsynaptic potentials of central neurons, capturing fine-grained changes in cortical excitability with high temporal resolution. In contrast, peripheral indicators such as heart rate and electrodermal activity undergo multi-level integration through the autonomic nervous system (e.g., sympathetic ganglia, adrenal medulla, heart/sweat glands), during which the signal is smoothed and is also susceptible to physiological noise such as respiration, body temperature, and minor movements. Therefore, peripheral indicators may fail to detect significant differences due to insufficient signal-to-noise ratio. (2) EEG and peripheral indicators reflect stress responses operating on different time scales and via different neural pathways. EEG measures real-time cortical electrical activity (millisecond level), which is more directly related to the psychological and cognitive processing of stress. In contrast, heart rate variability, electrodermal activity, and cortisol reflect autonomic tone (seconds to minutes) and HPA axis hormone secretion (minutes to hours), respectively, involving more downstream effectors. Sex differences may primarily exist at the stage of early cognitive appraisal and cortical processing of stress, and may be partially “buffered” at the output level of peripheral effectors—potentially explaining why no significant differences were found in indicators such as heart rate variability. (3) Statistical power considerations. The sample size of this study (24 per group) was determined based on a medium effect size, but may still be insufficient for detecting interaction effects. Some sex differences that did not reach statistical significance (e.g., cortisol, electrodermal response) may stem from limited statistical power rather than the absence of a true effect. Future studies should recruit larger samples to detect smaller effect sizes of sex differences.

### 4.4. Limitations

This study has several limitations that should be acknowledged. First, this study only adopted a within-subject pre–post comparison design and did not include a condition control group. Therefore, the differences between the UDST and the SECPT cannot be uniquely attributed to “uncertainty,” because the two paradigms differ not only in the dimension of uncertainty but also in the type of stressor. Future research should refine the control conditions: on one hand, a “certain-drop group” (drop at fixed time points) and a “standing control group” (standing without any drop) could be added to isolate the independent contribution of uncertainty; on the other hand, multiple drop heights could be set to examine the modulatory effect of this physical parameter on stress intensity. Second, the sample size of this study was limited, and the participants were restricted to college students. Although the sample size was determined based on a medium effect size, larger samples may be needed for complex interactions, and some non-significant sex differences may be due to insufficient statistical power rather than the absence of true effects. Future studies should recruit larger samples and expand the types of participant populations to examine possible sex differences. Finally, although the UDST better simulates the pervasive uncertainty characteristic of real-world environments compared to traditional certain-stress paradigms (e.g., SECPT), it remains, in essence, a controlled laboratory model and cannot fully replicate the full complexity of real stress environments; its ecological validity is still limited. Future research could combine virtual reality technology to construct immersive, dynamically changing uncertain stress scenarios.

## 5. Conclusions

(1) UDST effectively induced psychophysiological stress responses in individuals, demonstrating significantly superior induction efficacy compared to SECPT.
(2) The two stress methods evoked distinct neural oscillatory patterns: UDST triggered an “exogenous vigilance mode” characterized by enhanced high-frequency activity, while SECPT elicited an “interoceptive focusing mode” characterized by suppressed low-frequency activity.
(3) Females displayed stronger stress reactivity than males in terms of heart rate and neural oscillations.

## Figures and Tables

**Table 1:** Heart rate variability indicators.

	Indicator	Meaning	Unit	Interpretation
Time Domain	RR	Interval between normal sinus heartbeats	ms	Overall HRV indicator
	SDNN	Standard deviation of normal sinus heartbeats	ms	Negatively correlated with sympathetic activity
	RMSSD	Root mean square of successive RR interval differences	ms	Positively correlated with parasympathetic activity
	PNN50	Percentage of successive RR intervals differing by >50 ms	%	Positively correlated with parasympathetic activity
Frequency Domain	HF norm	Normalized high-frequency power		Proportion of high-frequency power

**Table 2:** Descriptive statistics and ANOVA results for heart rate variability indicators (*M* ± *SD*).

Indicator	Gender	SECPT		UDST		Gender	Condition	Time	Condition × Time
		T0	T1	T0	T1	*p*	*p*	*p*	*p*
RR	Male	859.19 ± 114.82	833.51 ± 106.21	888.24 ± 119.29	722.33 ± 94.59	0.027	<0.001	<0.001	<0.001
	Female	802.18 ± 125.39	759.79 ± 118.92	818.98 ± 103.02	658.52 ± 90.06				
RMSSD	Male	41.48 ± 19.99	46.53 ± 20.05	43.88 ± 18.42	34.46 ± 17.10	0.723	0.175	0.067	0.008
	Female	44.80 ± 21.93	42.43 ± 19.12	45.74 ± 18.28	40.00 ± 19.33				
PNN50	Male	20.24 ± 16.04	22.32 ± 14.63	24.34 ± 19.02	12.26 ± 12.70	0.615	0.054	<0.001	<0.001
	Female	25.03 ± 21.32	22.09 ± 14.83	25.70 ± 17.99	14.35 ± 11.67				
HF norm	Male	43.85 ± 23.11	42.70 ± 15.56	49.30 ± 21.31	33.81 ± 15.53	0.001	0.121	<0.001	<0.001
	Female	62.35 ± 17.48	53.19 ± 19.52	66.67 ± 12.87	40.04 ± 16.08				

Note: Interactions involving Gender × Time and Gender × Time × Condition did not reach statistical significance and are therefore not included in the table.

## Data Availability

The data presented in this study are available on request from the corresponding author. The data are not publicly available as they are subject to privacy restrictions and the related experiments have not been fully completed; therefore, the data are not currently appropriate for public dis-closure.
